# Circulating MicroRNA Levels Indicate Platelet and Leukocyte Activation in Endotoxemia Despite Platelet P2Y_12_ Inhibition

**DOI:** 10.3390/ijms21082897

**Published:** 2020-04-21

**Authors:** Aitana Braza-Boïls, Temo Barwari, Clemens Gutmann, Mark R. Thomas, Heather M. Judge, Abhishek Joshi, Raimund Pechlaner, Manu Shankar-Hari, Ramzi A. Ajjan, Ian Sabroe, Robert F. Storey, Manuel Mayr

**Affiliations:** 1 King’s British Heart Foundation Centre, King’s College London, London SE5 9NU, UK; aitana_braza@iislafe.es (A.B.-B.); temo.barwari@kcl.ac.uk (T.B.); clemens.gutmann@kcl.ac.uk (C.G.); abhishek.joshi@kcl.ac.uk (A.J.); 2 Health Research Institute La Fe, 46026 Valencia, Spain; 3 Institute of Cardiovascular Sciences, University of Birmingham, Birmingham B15 2TT, UK; m.r.thomas@bham.ac.uk; 4 Department of Infection, Immunity and Cardiovascular Disease, University of Sheffield, Sheffield S10 2RX, UK; h.judge@sheffield.ac.uk (H.M.J.); i.sabroe@sheffield.ac.uk (I.S.); r.f.storey@sheffield.ac.uk (R.F.S.); 5 Department of Neurology, Medical University Innsbruck, Innsbruck 6020, Austria; raimund.pechlaner@i-med.ac.at; 6 Critical Care Medicine, Guy’s and St Thomas’ NHS Foundation Trust, London SE1 7EH, UK; manu.shankar-hari@kcl.ac.uk; 7 Leeds Institute of Cardiovascular and Metabolic Medicine, University of Leeds, Leeds LS2 9JT, UK; r.ajjan@leeds.ac.uk

**Keywords:** biomarker, microRNA, antiplatelet therapy, sepsis

## Abstract

There is evidence for the effects of platelet inhibition on innate immune activation. Circulating microRNAs (miRNAs) have been implicated as markers of platelet and leukocyte activation. In the present study, we assessed the effects of P2Y_12_ inhibitors on platelet and leukocyte miRNAs during endotoxemia. Healthy volunteers were randomly assigned to receive oral ticagrelor (*n* = 10), clopidogrel (*n* = 8) or no drug (*n* = 8) for one week, followed by an intravenous bolus of 2 ng/kg endotoxin. Serum was collected at baseline, after one week of antiplatelet treatment and 6 and 24 h after endotoxin administration. MiRNAs were screened using LNA-based qPCR, followed by TaqMan-qPCR validation of candidates. Clinical validation was performed in 41 sepsis patients. Platelet-enriched miR-197, miR-223 and miR-223* were decreased in volunteers following antiplatelet therapy. Endotoxin increased platelet miRNAs, whilst the opposite effect was seen for leukocyte-enriched miR-150. Neither of these endotoxin-mediated effects were altered by P2Y_12_ inhibitors. Sepsis patients with fatal outcomes (*n* = 12) had reduced miR-150 levels compared with survivors (*n* = 29). In conclusion, we show that miR-150 is downregulated in experimental endotoxemia and can predict survival in sepsis but is unaffected by P2Y_12_ inhibition. While P2Y_12_ inhibition reduces platelet-associated miRNAs in healthy volunteers, it fails to attenuate the response of platelet miRNAs to endotoxemia.

## 1. Introduction

Sepsis affects around 31.5 million people per year globally, of which approximately 5.3 million die [1]. With a median of the mean hospital-wide cost of sepsis per patient of $32,421, as systematically reviewed in 2017, it also represents the most expensive condition in US hospitals [2]. Sepsis is characterized by an excessive immune response to infection that leads to organ dysfunction [3]. As a consequence of the systemic presence of inflammatory stimuli, platelets are activated on a large scale. This further aggravates septic coagulation and inflammatory reactions, potentially leading to disseminated intravascular coagulation [4]. Although the mechanisms are not fully understood, increased platelet reactivity might also contribute to the 18-fold risk increase of myocardial infarction or stroke within 30 days of bacteremia [5]. The question whether (and which) platelet inhibitors are able to attenuate this detrimental process is therefore highly relevant. This has been addressed in pre-clinical sepsis models as well as a few, mostly retrospective clinical studies. Some of these studies point to the direction that sepsis patients on antiplatelet therapy have survival benefits [6,7,8], which are attributed to the drug’s anti-inflammatory properties in addition to their antiaggregatory effects, as determined by experimental studies [9,10,11]. Other studies did not confirm benefits on severity and outcome, however, and the hypercoagulable state in sepsis remains difficult to manage [12,13,14].

One reason for limited success of antiplatelet drugs is high on-treatment platelet reactivity during sepsis. In fact, recent data suggests that sepsis promotes platelet activation despite treatment with P2Y_12_ inhibitors, which are common antiplatelet drugs used in the secondary prevention of arterial thrombotic diseases [15,16,17]. Currently, there are no diagnostic tests that are able to inform treatment decisions and predict outcomes based on measurements of platelet reactivity in sepsis patients. Indeed, early identification of the best individualized treatment strategy remains challenging in sepsis because clinical signs and laboratory parameters are nonspecific [18]. It is therefore important to find novel markers that can be used in combination with established clinical scores and laboratory parameters. MiRNAs are attracting interest as potential biomarkers. The main biological function of these small RNAs (~22 nucleotides in length) is to repress protein synthesis. Most miRNAs are ubiquitously expressed, but a small subset is cell-specific and can be dysregulated in disease [19]. Their stable detectability in cell-free serum or plasma led to their investigation as biomarkers for various conditions, including immune cell activation in sepsis [20] and response of platelets to antiplatelet therapy [21]. However, the identification of a suitable miRNA biomarker in sepsis has been complicated by the numerous confounders present in such critically ill patients. Comorbidity, comedication, source and type of infection can affect the extracellular miRNome and lead to high interindividual variations in the immune response. This contributes to the conflicting evidence in the literature with regards to changes of sepsis-related miRNAs [20].

To minimize the impact of clinical confounders and preanalytical variation, we performed serum miRNA profiling in an experimental endotoxemia model, with volunteers receiving the P2Y_12_ inhibitors ticagrelor, clopidogrel or no drug [22]. Findings were then validated in a cohort of sepsis patients.

## 2. Results

### 2.1. Effect of Antiplatelet Therapy on Circulating MiRNAs

**Figure 1:**
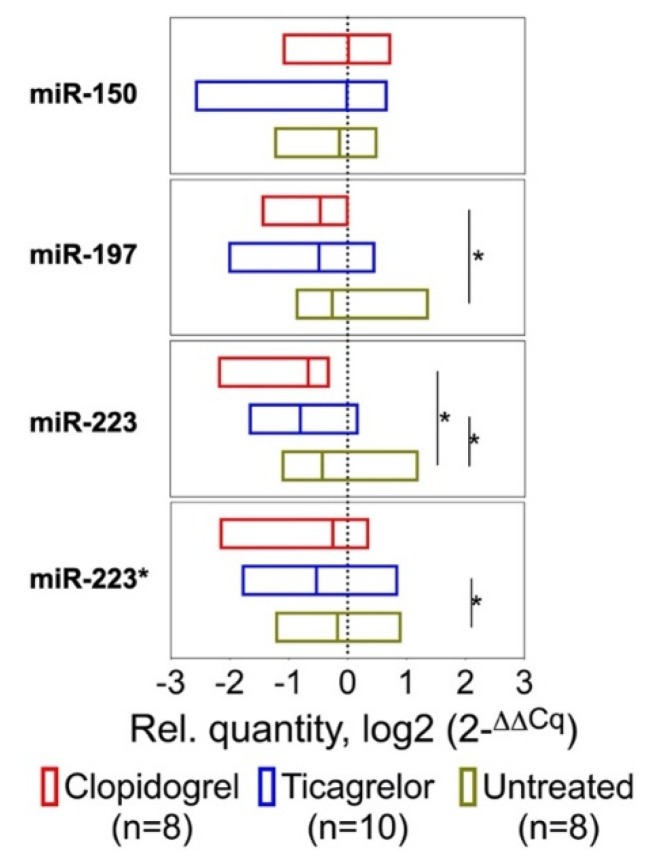
Circulating miRNA levels after antiplatelet therapy. Levels of platelet-enriched miR-197, miR-223 and miR-223* were significantly lower after one week of treatment with either ticagrelor or clopidogrel, compared with the untreated control group. Leukocyte-enriched miR-150 was not affected. Bars and lines represent range and median. Wilcoxon’s signed-rank test was used for statistical comparison. * denotes *p*-value < 0.05.

To identify circulating miRNAs that are responsive to antiplatelet therapy and can serve as markers of platelet activation, miRNA levels were profiled by TaqMan-based qPCR analysis in serum of healthy volunteers, who were randomly assigned to receive oral ticagrelor (180 mg loading dose, followed by 90 mg maintenance dose twice daily), oral clopidogrel (300 mg loading dose, followed by 75 mg twice daily) or no treatment for one week. Platelet-enriched miR-197, miR-223 and miR-223* were significantly downregulated after treatment with clopidogrel or ticagrelor (Figure 1, Table S1) [23]. In contrast, antiplatelet therapy had no effect on leukocyte-enriched miR-150 [24].

### 2.2. Effect of Endotoxemia on Circulating MiRNAs

**Figure 2:**
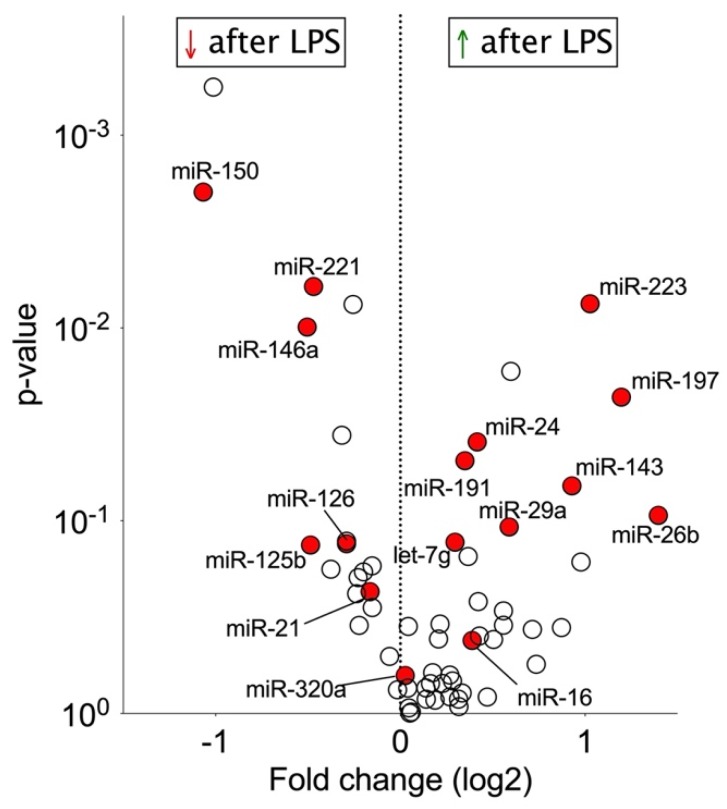
Circulating miRNA levels after endotoxemia. Volcano plot representing log2 fold change of miRNA levels 6 h after endotoxin infusion in healthy volunteers without antiplatelet therapy (*n* = 6). Leukocyte-enriched miR-150 showed strongest fall in abundance, while platelet-enriched miR-197 and miR-223 showed the strongest rise. MiRNAs highlighted in red were selected for qPCR analysis in the entire cohort. Student’s t-tests were used to calculate *p*-values.

Next, miRNA profiling was performed in an experimental human model of low-dose endotoxemia [22]. Healthy volunteers from the control group without antiplatelet therapy (*n* = 6) received an intravenous bolus of 2 ng/kg endotoxin. With this standardized approach, common clinical confounders and preanalytical variation in sepsis patients can be avoided. When miRNA levels were compared before and 6 h after endotoxin administration, leukocyte-enriched miR-150 was found to have the strongest decrease (Figure 2, Table S2) [24]. In contrast, miRNAs previously implicated as markers of platelet activation (miR-197, miR-223, miR-26b, miR-191, miR-24; [23,25,26]) showed higher levels after endotoxin treatment.

### 2.3. Effect of Antiplatelet Therapy on Circulating MiRNAs in Endotoxemia

**Figure 3:**
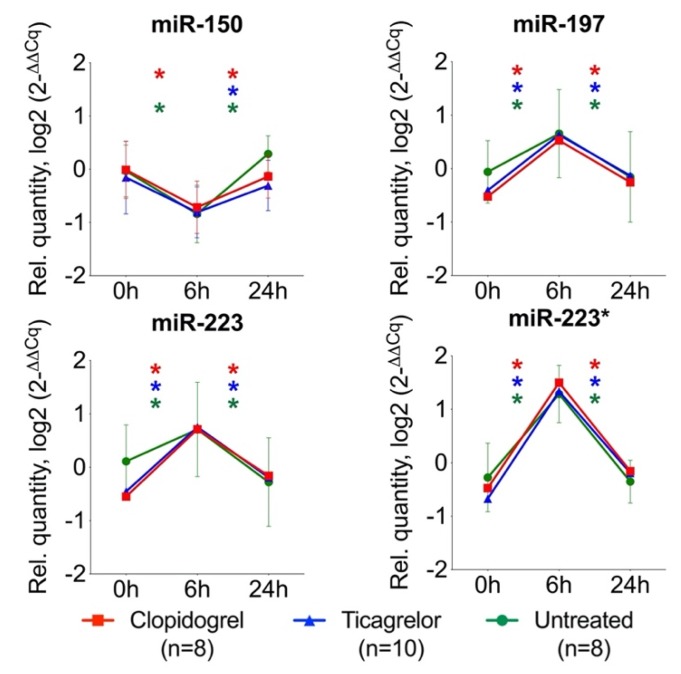
Effect of antiplatelet therapy on miRNA levels in endotoxemia. Endotoxin administration in volunteers markedly reduced levels of leukocyte-enriched miR-150 at 6 h after endotoxin administration (6 h), while platelet-enriched miR-197, miR-223 and miR-223* showed an increase. Neither of these effects of endotoxin were altered by treatment with clopidogrel (red) or ticagrelor (blue), compared to the untreated group (green). After 24 h, miRNAs returned to baseline levels (0 h). Data is represented as the geometrical mean with the 95% confidence interval. Statistical testing was performed using Wilcoxon’s signed rank test for consecutive timepoints within each treatment group. *p*-values were adjusted for multiple testing by the Benjamini–Hochberg method. * denotes False Discovery Rate-adjusted *p*-value <0.05.

Healthy volunteers were randomly assigned to receive oral ticagrelor (*n* = 10), clopidogrel (*n* = 8) or no treatment (*n* = 8) for one week, followed by an intravenous bolus of 2ng/kg endotoxin. Twenty-one candidate miRNAs that were differentially regulated in endotoxemia (Figure 2) and had evidence for enrichment in platelets or leukocytes were selected. These miRNAs were then analyzed by TaqMan-based qPCR in the entire cohort. At 6 h after endotoxin administration, levels of leukocyte-enriched miR-150 were markedly reduced, confirming findings from the previous screen (Figure 3, Table S3). For platelet-enriched miRNAs, an increase at 6 h after endotoxin administration was observed. After 24 h, all miRNAs returned to baseline. Neither of these effects of endotoxin were altered by pretreatment with clopidogrel or ticagrelor, suggesting that antiplatelet therapy does not attenuate platelet activation or the innate immunity response in this model of low-dose endotoxemia.

### 2.4. Circulating MiR-150 Levels Are Lower in Sepsis Patients with Fatal Outcome Than in Survivors

**Figure 4:**
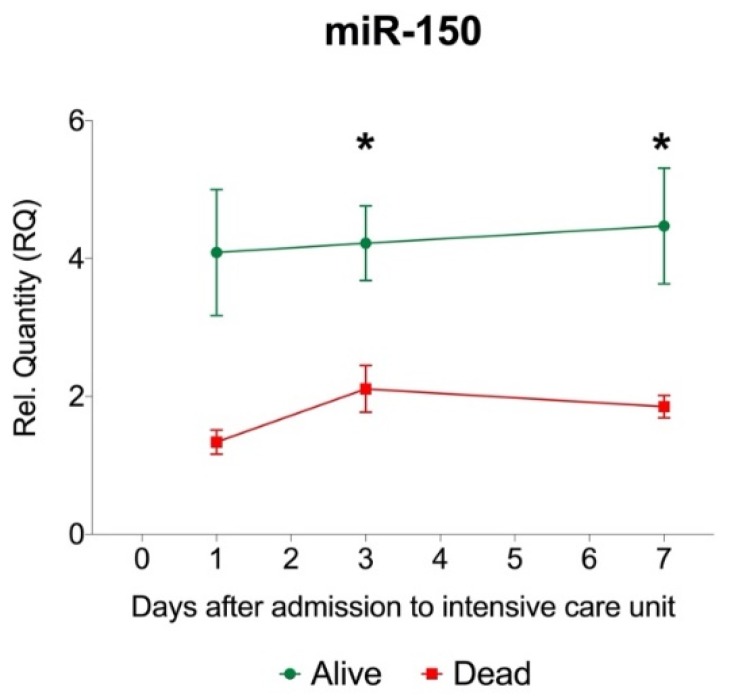
Levels of miR-150 in sepsis patients. Sepsis patients with fatal outcome (*n* = 12) had significantly lower miR-150 levels in serum at day 3 and day 7 compared with sepsis survivors (*n* = 29). Graph depicts mean ± standard error of the mean. Two-way ANOVA with Dunnett’s multiple comparisons test was used for statistical comparison. * denotes *p*-value <0.05.

To validate the findings of our endotoxemia model in a clinical context, selected platelet- and leukocyte-enriched serum miRNAs were measured in 41 sepsis patients at day 1, 3 and 7 after admission to the intensive care unit (Table S4). As previously reported [27,28], miR-150 levels were lower in non-survivors compared with survivors (Figure 4, Table S5). In contrast, levels of platelet-related miRNAs miR-197 and miR-223 did not differ between sepsis survivors and non-survivors (Table S5).

## 3. Discussion

This study aimed to identify miRNA biomarkers for platelet and immune cell activation in endotoxemia. To define the circulating miRNA response to endotoxemia without common confounders such as medication and co-morbidities in critically-ill sepsis patients, we employed an experimental low-dose endotoxemia model in combination with P2Y_12_ inhibitor treatment in healthy volunteers. Findings were subsequently applied to a cohort of sepsis patients [22].

The three miRNAs responsive to P2Y_12_ inhibition (miR-197, miR-223 and miR-223*) have previously been described as being enriched in platelets [23]. Also, a crossover study including 56 patients with type 2 diabetes mellitus found plasma levels of miR-197 and miR-223 alongside miR-191 and miR-24 to be lower in diabetic patients on the P2Y_12_ inhibitor prasugrel, compared with aspirin [26]. We have previously reported a decrease of these platelet-related miRNAs in plasma of healthy volunteers upon treatment with P2Y_12_ inhibitors [23]. In 121 patients with a history of acute coronary syndrome, plasma levels of miR-223, miR-197, miR-191 and miR-24 showed significant positive correlations with the vasodilator-stimulated phosphoprotein phosphorylation assay but not light transmittance aggregometry tests after 30 days of dual antiplatelet therapy [29]. In contrast to our results, studies using platelet-rich or platelet-poor plasma from patients with acute coronary syndrome found miR-223 levels to be decreased in patients with a very low response to P2Y_12_ inhibitors, compared with normal responders [30,31,32]. Differences in sample preparation and normalization, as well as potential interference of heparin [33], can all substantially affect measurements of circulating miRNAs [21]. Alternatively, low miR-223 levels in acute coronary syndrome patients with a low response to P2Y_12_ inhibitors might be the consequence of reduced expression in platelets of this patient subgroup. MiR-223 is known to target P2Y_12_ mRNA and lower levels may therefore convey increased resistance to P2Y_12_ inhibition. Reduced expression of miR-223 in platelets has also been shown in diabetes; which is a strong risk factor for coronary artery disease and can be associated with high on-treatment platelet reactivity [34,35].

In contrast to platelet-derived miR-197, miR-223 and miR-223*, levels of leukocyte-enriched miR-150 were unaffected by antiplatelet therapy [24]. Experimental endotoxemia led to a rise of platelet-associated miRNAs in the circulation, indicating endotoxin-mediated platelet activation. The latter is mediated by platelet toll-like receptor 4 [36,37,38,39]. Facilitated by sCD14 derived from plasma [39], endotoxin binds to this receptor and initiates a signaling cascade that involves the adaptor protein MyD88, resulting in activation of the nitric oxide and cyclic guanosine monophosphate-dependent protein kinase pathway. This is sufficient to induce secretion of dense and α-granules but does not induce platelet aggregation, as determined by ex vivo experiments [36,37,38]. Instead, endotoxin sensitizes and potentiates the aggregation response to subthreshold concentrations of common platelet agonists such as adenosine diphosphate, collagen, glycoprotein VI collagen receptor agonists, thrombin and thromboxane [36,37,38]. There is also evidence that platelet mRNA levels of interleukin-1β, tissue factor and αIIb protein are increased and translated under septic conditions, further contributing to the prothrombotic state [40,41,42].

The rise of platelet-associated miR-197, miR-223 and miR-223* levels observed after low-dose endotoxemia was unaffected by treatment with clopidogrel or ticagrelor, suggesting release of platelet-associated miRNAs despite pharmacological P2Y_12_ inhibition. One explanation for high on-treatment platelet reactivity during sepsis is the dysregulation of hepatic cytochrome P450 enzymes upon endotoxemia [43], which are responsible for activation of the prodrug clopidogrel. Another possibility is that high on-treatment platelet reactivity is mediated by platelets that escape pharmacological therapy when they are formed at the nadir of drug bioavailability [44]. However, both mechanisms are unlikely since clopidogrel and ticagrelor showed indistinguishable effects on circulating miRNAs; despite ticagrelor being an allosteric antagonist that does not require enzymatic activation and is present continuously in the plasma at therapeutic concentrations during treatment [45]. Instead, there is evidence that P2Y_12_ inhibitors insufficiently reduce platelet reactivity during sepsis [15,16,17]. Patients on clopidogrel upon sepsis onset showed high on-treatment platelet reactivity in a prospective observational study, as determined by the VerifyNow point-of-care P2Y_12_ assay [15]. Similarly, healthy volunteers on prasugrel showed reduced antiplatelet effects due to the increased release of von Willebrand factor during experimental endotoxemia [16]. A beneficial role of P2Y_12_ inhibitors beyond their intended effects on platelets appears to be the reduction of systemic inflammation. This has been shown in sepsis models conducted in animals [46,47] as well as humans [48]. In a human experimental endotoxemia model conducted by Thomas et al. [48], clopidogrel and ticagrelor were able to reduce peak levels of D-dimer and major proinflammatory cytokines, including interleukin-6, tumor necrosis factor-α and monocyte chemoattractant protein-1. In contrast to clopidogrel, ticagrelor also reduced interleukin-8 and growth colony-stimulating factor levels, increased interleukin-10 levels and reduced platelet-monocyte-, but not platelet-neutrophil aggregation. In line with this observation, another study confirmed decreased release of pro-inflammatory cytokines in blood from ticagrelor-treated volunteers when exposed to endotoxin ex vivo [49]. Platelet-monocyte aggregates are known to amplify monocyte release of proinflammatory cytokines that are responsible for the excessive immune response in sepsis, and their prevention could therefore be beneficial [50,51]. Consistent with the absent response of platelet-neutrophil aggregation upon P2Y_12_ inhibition in the study by Thomas et al. [48], another human experimental endotoxemia study by Schoergenhofer et al. [17] found no influence of prasugrel on circulating levels of histone-DNA complexes. The latter serve as surrogates of extracellular traps derived from neutrophils (NETs), which can be formed following platelet toll-like receptor 4-mediated platelet-neutrophil aggregation [52]. These structures were initially described for their importance in host defense and are now being increasingly recognized for their prothrombotic role [53].

In contrast to the increase of platelet-associated miRNAs, leukocyte-enriched miR-150 was markedly reduced in experimental endotoxemia and lower in sepsis patients with fatal outcomes, compared with survivors. Clopidogrel and ticagrelor had no influence on miR-150 levels in our model, suggesting that P2Y_12_ inhibition does not attenuate its reduction during low-dose endotoxemia. In a previous study, intracellular miR-150 levels were found to be decreased in leukocytes upon human experimental endotoxemia [24]. Its downregulation in the circulation and negative correlation with survival in sepsis patients has also been reported before [27,28,54]. In a pilot study with 17 sepsis patients and 32 healthy controls, miR-150 levels were reduced and correlated with disease severity assessed by the sequential organ failure assessment (SOFA) score [28]. Here, miR-150 also negatively correlated with levels pro-inflammatory cytokines interleukin-18, tumor necrosis factor-α and interleukin-6, but not leukocyte numbers [28]. This was reproduced in a larger study, which included a training cohort and independent validation cohort [54]. In the latter, miR-150 levels were downregulated in patients with sepsis compared with individuals affected by systemic inflammatory response syndrome and healthy volunteers. In another study, miR-150 levels predicted survival in a cohort of 223 critically ill patients, of which 138 fulfilled sepsis criteria [27]. Reduced miR-150 levels were also shown in a murine sepsis model [55]. Functionally, miR-150 controls the transcription factor transcriptional activator Myb, which affects the development and immune response of lymphocytes [56]. In monocytes, miR-150 regulates the generation of non-classical monocyte subsets, with miR-150 being upregulated in non-classical monocytes and downregulated in classical monocytes [57]. Increased differentiation of monocytes towards the non-classical phenotype has been associated with survival in sepsis [58]. In light of our finding that miR-150 is decreased in serum upon experimental human endotoxemia, it appears that miR-150 is a robust sepsis marker.

Our study has quantified miRNAs in cell-free serum, which is characterized by high RNase activity [59]. The stable detectability of circulating miRNAs has been attributed to their protection by small and large extracellular vesicles [60,61,62] and/or protein complexes [62,63,64]. Recently, it was reported by Linhares-Lacerda et al. [53] that miRNAs can also be carried by NETs. In fact, NET formation could be a relevant mechanism by which miR-150 is released from neutrophils. In sepsis patients, platelet toll-like receptor 4 has been shown to induce platelet-neutrophil aggregation and subsequent NET-formation [52]. According to the findings by Schoergenhofer et al. [17] and Thomas et al. [48], P2Y_12_ inhibition reduced systemic inflammation but not platelet-neutrophil aggregation and NET markers in humans given a single bolus of endotoxin; a possible NET-dependent miR-150 release could explain why this miRNA is such a robust marker of endotoxemia but is not affected by the anti-inflammatory effects of P2Y_12_ inhibitors. The response to antiplatelet treatment during endotoxemia, however, might differ for other antiplatelet drugs. There is limited evidence for aspirin to inhibit endotoxin-mediated platelet activation ex vivo [37]. A recent meta-analysis by Ouyang et al. [65] has shown that for all but one study [6], the positive effect of different antiplatelet drugs on sepsis mortality is lost when patients on aspirin are excluded. Most studies, however, are retrospective rather than interventional, and there is currently no consensus on the benefit of antiplatelet therapy in sepsis.

In summary, our findings indicate that miR-150 is a robust marker of sepsis mortality, which is in line with previous studies [27,28,54]. Reduced miR-150 levels were observed in sepsis patients and in experimental endotoxemia, confirming that endotoxin is sufficient to induce this response, at least in healthy volunteers. The P2Y_12_ inhibitors clopidogrel and ticagrelor do not alter the miR-150 response, nor do they attenuate the endotoxin-induced release of platelet-associated miRNAs (miR-197, miR-223 and miR-223*). While these platelet-associated miRNAs are responsive to P2Y_12_ inhibition in the absence of endotoxemia, their response during sepsis does not allow discrimination between survivors and non-survivors. Further studies are needed to clarify whether certain miRNAs have the potential to improve diagnosis, prognosis assessment and inform treatment decisions in sepsis.

## 4. Materials and Methods

### 4.1. Study Design and Participant Characteristics

**Figure 5:**
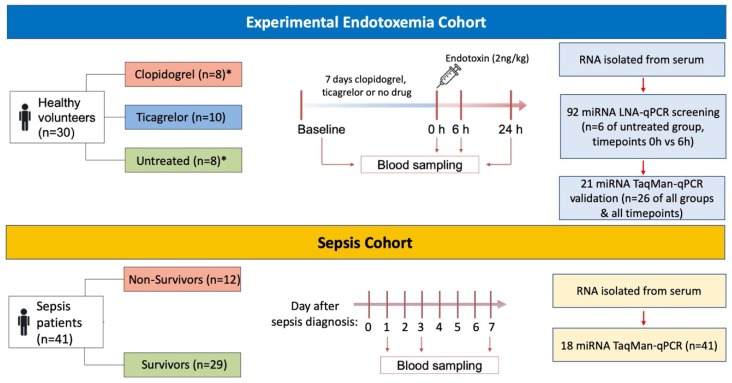
Schematic of experimental design. * Samples of 2 volunteers from the clopidogrel group and from the untreated group had to be excluded due to hemolysis.

Two independent cohorts were included in the study (Figure 5). The first cohort consisted of 30 healthy volunteers (median age 22 years, all male). A detailed description of the study population can be found here [48]. All participants were randomly assigned to three experimental groups according to the antiplatelet therapy they received. In brief, group A received oral ticagrelor (180 mg loading dose, followed by a maintenance dose of 90 mg twice daily for 7 days). Group B received oral clopidogrel (300 mg loading dose, followed by a maintenance dose of 75 mg once daily for 7 days). Group C did not receive any treatment and served as a control group. All participants then received an intravenous bolus of 2 ng/kg endotoxin. Serum was collected by venipuncture into serum separator tubes at baseline (before starting antiplatelet therapy); after one week of antiplatelet therapy (before endotoxin bolus); and 6 and 24 h after endotoxin infusion. Samples of 2 volunteers from group B and samples of 2 volunteers from group C were excluded from miRNA analysis due to hemolysis. The study was approved by the Sheffield Research Ethics Committee (UK) on 7th of February 2013 (REC reference 13/YH/0005). Participants provided written informed consent [48].

The second cohort consisted of 41 sepsis patients (median age 64 years, 61% male) recruited in an intensive care unit. Clinical management of all patients in the sepsis cohort was at the discretion of the attending physicians. A detailed description of the study population can be found in Supplementary Table S4. Serum was collected by venipuncture into serum separator tubes at days 1, 3 and 7 after enrolment. The study was approved by an institutional review board (REC reference 12/LO/0326). Written informed consent was obtained directly from patients (if mentally competent), or from the next of kin. The consent procedure was then completed with retrospective consent [66].

### 4.2. RNA Isolation

Total RNA was extracted from 100 μL serum using the miRNeasy Mini kit (Qiagen, Hilden, Germany, #217004) according to the manufacturer’s recommendations, with minor modifications as described previously [67]. At the first step of the isolation, 1.25 μL RNA from MS2 bacteriophage (Roche, Basel, Switzerland, #10165948001) and 1:2000 diluted Cel-miR-39-3p mimic (Qiagen, #219610) were spiked-in as carrier and exogenous control, respectively. Total RNA was eluted in 35 μL of nuclease-free water.

### 4.3. Reverse Transcription and Real Time Quantitative Polymerase Chain Reaction (qPCR)

For the screening experiment that was conducted in healthy volunteers before and 6h after endotoxin administration, Locked Nucleic Acid (LNA)-based qPCR was used (92 miRNAs; Supplementary Table S2). Reverse transcription was performed with the miRCURY LNA™ Universal RT microRNA PCR panel (Exiqon, Vedbaek, Denmark, #203301) following the manufacturer’s instructions, using a Veriti Thermal Cycler (Applied Biosystems, Foster City, USA). qPCR was then performed with the miRCURY Ready-to-Use PCR, Human panel I + II V1.M platform (Exiqon, Vedbaek, Denmark, #339322) following the manufacturer’s instructions, using an Applied Biosystems Viia 7 thermocycler. Screening data was normalized based on the 2^−ΔΔCq^ method [68], using the average Cq of all measured transcripts for ΔCq and an interplate calibrator consisting of an RNA pool of all samples for ΔΔCq.

For the validation experiment in the whole cohort of healthy volunteers (21 miRNAs; Supplementary Tables S1 and S3) and the miRNA measurements in sepsis patients (18 miRNAs; Supplementary Table S5), TaqMan-based qPCR was used. Reverse transcription was performed using Megaplex RT primers (Human Pool A v2.; Life Technologies, Darmstadt, Germany, #4399966) and the TaqMan MicroRNA RT kit (Life Technologies, Darmstadt, Germany, #4366596) according to the manufacturer’s protocol. cDNA was then pre-amplified using Megaplex PreAmp Primers (Human Pool A v2.1, Life Technologies, Darmstadt, Germany, #4399233) and TaqMan PreAmp Mastermix (Life Technologies, Darmstadt, Germany, #4488593). Pre-amplification product was diluted 1:18 and TaqMan Universal PCR Master Mix No AmpErase UNG (Life Technologies, Darmstadt, Germany, #4324018) was used for qRT-PCR in an Applied Biosystems Viia 7 thermocycler. Validation data and data of sepsis patients was normalized based on the 2^−ΔΔCq^ method [68], using exogenous Cel-miR-39-3p for ΔCq and an interplate calibrator consisting of an RNA pool of all samples for ΔΔCq. 

### 4.4. Data Analysis

Statistical analysis and design of graphs was performed with R programming environment and GraphPad Prism (GraphPad Software Inc, San Diego, USA, Version 8). Statistical tests, measures of central tendency and variation are indicated in the respective result figures. A *p*-value of <0.05 was considered to denote statistical significance in all cases.

## 5. Patents

M.M. has filed and licensed patent applications on miRNAs as platelet biomarkers.

## Supplementary Materials

Supplementary materials can be found at https://www.mdpi.com/1422-0067/21/8/2897/s1. Table S1: List of 21 miRNAs measured by TaqMan-based qPCR in volunteers treated with ticagrelor (*n* = 8), clopidogrel (*n* = 10) or no drug (*n* = 8). Table S2: Experimental endotoxemia screening experiment. Table S3: List of 21 miRNAs measured by TaqMan-based qPCR in volunteers treated with ticagrelor (*n* = 10), clopidogrel (*n* = 8) or no drug (*n* = 8) before (0 h) and after (6 h, 24 h) endotoxin administration. Table S4: Clinical characteristics of the cohort of sepsis patients. Table S5: List of 18 miRNAs measured by TaqMan-based qPCR in sepsis patients at day 1 (d1), day 3 (d3) and day 7 (d7) after admission to the intensive care unit.
