# Supplementary material for: Circulating MicroRNA Levels Indicate Platelet and Leukocyte Activation in Endotoxemia Despite Platelet P2Y12 Inhibition
Source: Int J Mol Sci. 2020 Apr 21;21(8):2897. doi: 10.3390/ijms21082897 (PMC7215420; doi:10.3390/ijms21082897)
Supplement: Supplementary file 1 [file ijms-21-02897-s001.pdf]

Suppl. Table 1. List of 21 miRNAs measured by TaqMan-based qPCR in volunteers treated with ticagrelor (n = 8), clopidogrel (n = 10) or no drug (n = 8). APT denotes antiplatelet therapy, RQ: relative quantity; green colour indicates an increase and red a decrease after 1-week follow-up.

miRNA	Treatment	Median RQ - before APT	Median RQ - after APT	Median Difference	Difference in Medians	Test Statistic	Effect	P-value
hsa-let7g	Clopidogrel	0.36	0.23	-0.10	-0.13	1.12	0.40	0.31
	Ticagrelor	0.57	0.41	-0.10	-0.16	1.07	0.34	0.32
	Untreated	0.33	0.48	0.08	0.14	0.28	0.10	0.84
hsa-miR-125b	Clopidogrel	0.43	0.71	0.39	0.27	-1.54	-0.54	0.15
	Ticagrelor	0.51	0.93	**0.51**	**0.42**	-2.09	-0.66	**0.04**
	Untreated	0.46	1.08	**0.52**	**0.62**	-2.10	-0.74	**0.04**
hsa-miR-126	Clopidogrel	0.65	0.54	-0.12	-0.11	1.12	0.40	0.31
	Ticagrelor	0.78	0.58	-0.11	-0.20	0.36	0.11	0.77
	Untreated	0.57	0.67	-0.03	0.10	0.42	0.15	0.74
hsa-miR-126STAR	Clopidogrel	9.74	9.10	1.99	-0.64	-0.28	-0.10	0.84
	Ticagrelor	9.45	9.61	-0.19	0.16	0.66	0.21	0.56
	Untreated	8.29	9.74	0.78	1.45	-0.56	-0.20	0.64
hsa-miR-127-3p	Clopidogrel	0.50	0.38	**-0.23**	**-0.13**	2.38	0.84	**0.02**
	Ticagrelor	0.95	0.57	**-0.39**	**-0.37**	2.40	0.76	**0.01**
	Untreated	0.84	0.70	-0.07	-0.13	0.68	0.26	0.58
hsa-miR-143	Clopidogrel	0.54	0.43	0.01	-0.11	-0.56	-0.20	0.64
	Ticagrelor	0.63	0.57	0.05	-0.05	-0.36	-0.11	0.77
	Untreated	0.49	0.60	-0.08	0.10	0.56	0.20	0.64
hsa-miR-146a	Clopidogrel	0.59	0.47	-0.08	-0.11	1.54	0.54	0.15
	Ticagrelor	0.53	0.44	-0.15	-0.09	1.89	0.60	0.06

	Untreated	0.50	0.50	-0.05	0.00	0.42	0.15	0.74
hsa-miR-150	Clopidogrel	0.33	0.36	0.02	0.02	-0.14	-0.05	0.95
	Ticagrelor	0.41	0.42	0.01	0.01	-0.15	-0.05	0.92
	Untreated	0.37	0.39	-0.02	0.02	-0.28	-0.10	0.84
hsa-miR-16	Clopidogrel	0.27	0.46	0.13	0.19	-1.54	-0.54	0.15
	Ticagrelor	0.37	0.27	-0.01	-0.10	0.66	0.21	0.56
	Untreated	0.23	0.23	-0.01	0.00	0.00	0.00	1.00
hsa-miR-191	Clopidogrel	0.49	0.37	-0.12	-0.11	1.68	0.59	0.11
	Ticagrelor	0.48	0.38	**-0.08**	**-0.09**	2.29	0.73	**0.02**
	Untreated	0.43	0.40	-0.03	-0.03	0.70	0.25	0.55
hsa-miR-197	Clopidogrel	0.41	0.25	**-0.09**	**-0.16**	2.38	0.84	**0.02**
	Ticagrelor	0.40	0.29	-0.09	-0.11	1.78	0.56	0.08
	Untreated	0.38	0.35	-0.04	-0.03	0.98	0.35	0.38
hsa-miR-20b	Clopidogrel	0.51	0.54	0.12	0.03	-0.98	-0.35	0.38
	Ticagrelor	0.58	0.42	-0.06	-0.16	1.07	0.34	0.32
	Untreated	0.26	0.39	0.06	0.13	-0.84	-0.30	0.46
hsa-miR-21	Clopidogrel	0.94	0.87	-0.06	-0.07	0.42	0.15	0.74
	Ticagrelor	0.96	0.79	-0.08	-0.16	1.48	0.47	0.16
	Untreated	0.63	0.67	0.10	0.04	-0.84	-0.30	0.46
hsa-miR-221	Clopidogrel	0.88	0.66	-0.11	-0.22	1.40	0.50	0.20
	Ticagrelor	0.93	0.57	**-0.30**	**-0.37**	2.19	**0.69**	**0.03**
	Untreated	0.71	0.58	0.06	-0.12	0.00	0.00	1.00
hsa-miR-223	Clopidogrel	0.46	0.31	**-0.10**	**-0.15**	2.38	**0.84**	**0.02**
	Ticagrelor	0.48	0.36	**-0.14**	**-0.13**	2.19	**0.69**	**0.03**
	Untreated	0.51	0.55	-0.02	0.05	0.56	0.20	0.64
	Clopidogrel	5.34	3.68	-0.62	-1.65	0.98	0.35	0.38

hsa-miR-223STAR	Ticagrelor	6.33	5.23	**-1.30**	**-1.10**	2.09	0.66	**0.04**
	Untreated	6.76	5.35	-0.36	-1.41	0.70	0.25	0.55
hsa-miR-24	Clopidogrel	0.53	0.45	-0.11	-0.08	1.68	0.59	0.11
	Ticagrelor	0.61	0.50	**-0.13**	**-0.11**	1.99	0.63	**0.05**
	Untreated	0.57	0.51	0.02	-0.05	0.00	0.00	1.00
hsa-miR-26b	Clopidogrel	0.64	0.53	0.03	-0.10	-0.28	-0.10	0.84
	Ticagrelor	0.61	0.48	-0.04	-0.13	0.87	0.27	0.43
	Untreated	0.51	0.57	-0.03	0.06	0.14	0.05	0.95
hsa-miR-28-3p	Clopidogrel	0.62	0.35	-0.30	-0.27	1.68	0.59	0.11
	Ticagrelor	0.62	0.41	**-0.16**	**-0.21**	2.09	0.66	**0.04**
	Untreated	0.50	0.54	-0.06	0.04	0.84	0.30	0.46
hsa-miR-29a	Clopidogrel	0.61	0.49	-0.06	-0.12	1.26	0.45	0.25
	Ticagrelor	0.66	0.56	**-0.16**	**-0.10**	2.19	0.69	**0.03**
	Untreated	0.56	0.69	0.07	0.13	-0.70	-0.25	0.55
hsa-miR-320a	Clopidogrel	0.56	0.72	0.13	0.15	-1.40	-0.50	0.20
	Ticagrelor	0.57	0.49	-0.06	-0.08	1.27	0.40	0.23
	Untreated	0.38	0.40	-0.05	0.03	0.42	0.15	0.74

Suppl. Table 2. Experimental endotoxemia screening experiment. Log2 fold change and p-values of 92 miRNAs measured by LNA-based qPCR in healthy volunteers (n=6) before and 6h after endotoxin administration. Green colour indicates an increase and red a decrease after 6h.

miRNA	Fold change (log2)	P-value
**hsa-miR-152**	-1.013	**0.001**
**hsa-miR-150**	-1.067	**0.002**
**hsa-miR-221**	-0.470	**0.006**
**hsa-miR-223**	1.027	**0.007**
**hsa-miR-26a**	-0.256	**0.008**

**hsa-miR-146a**	-0.505	**0.010**
**hsa-miR-374a**	0.598	**0.017**
**hsa-miR-197**	1.196	**0.023**
**hsa-miR-126***	-0.317	**0.036**
**hsa-miR-24**	0.416	**0.039**
**hsa-miR-191**	0.350	**0.049**
hsa-miR-143	0.928	0.066
hsa-miR-26b	1.397	0.094
hsa-miR-29a	0.589	0.108
hsa-miR-192	-0.292	0.128
hsa-let-7g	0.296	0.129
hsa-miR-335	-0.457	0.131
hsa-miR-126	-0.292	0.132
hsa-miR-125b	-0.486	0.134
hsa-miR-125a-5p	0.367	0.153
hsa-miR-10a	-0.328	0.159
hsa-miR-423-5p	0.978	0.164
hsa-miR-30b	-0.153	0.171
hsa-let-7e	-0.376	0.179
hsa-miR-130a	-0.200	0.184
hsa-miR-107	-0.229	0.197
hsa-miR-21	-0.164	0.233
hsa-miR-199a-3p	-0.237	0.239
hsa-miR-20b	1.757	0.248
hsa-let-7b	0.422	0.262
hsa-miR-30c	-0.152	0.282

hsa-miR-15a	0.558	0.293
hsa-miR-155	-0.204	0.318
hsa-miR-17	0.022	0.323
hsa-miR-29b	0.215	0.343
hsa-miR-342-3p	0.558	0.348
hsa-miR-328	-0.223	0.349
hsa-miR-194	0.043	0.353
hsa-miR-590-5p	0.872	0.358
hsa-miR-186	0.715	0.366
hsa-miR-185	0.428	0.396
hsa-miR-139-5p	0.207	0.410
hsa-miR-25	0.505	0.413
hsa-miR-16	0.389	0.418
hsa-miR-331-3p	-0.144	0.431
hsa-miR-28-3p	1.051	0.444
hsa-miR-27a	-0.058	0.506
hsa-miR-363	0.736	0.555
hsa-miR-106a	0.175	0.614
hsa-miR-20a	0.267	0.632
hsa-miR-320a	0.027	0.635
hsa-miR-127-3p	0.544	0.636
hsa-miR-744	0.665	0.667
hsa-miR-140-5p	0.282	0.677
hsa-miR-27b	0.228	0.698
hsa-miR-195	-0.479	0.699
hsa-miR-19a	0.163	0.699

hsa-miR-532-3p	0.203	0.705
hsa-miR-454	-0.025	0.721
hsa-miR-222	0.138	0.737
hsa-miR-92a	0.040	0.739
hsa-miR-142-3p	-0.016	0.754
hsa-miR-660	0.334	0.783
hsa-miR-486-5p	0.269	0.819
hsa-miR-210	0.471	0.820
hsa-miR-19b	0.140	0.842
hsa-miR-18a	0.315	0.843
hsa-miR-122	0.188	0.853
hsa-miR-146b-5p	0.906	0.879
hsa-miR-451	0.317	0.919
hsa-miR-93	0.047	0.935
hsa-miR-130b	1.441	0.953
hsa-miR-484	0.059	0.981
hsa-miR-103	0.052	0.995
hsa-miR-342-5p	Undetermined	Undetermined
hsa-miR-202	Undetermined	Undetermined
hsa-miR-28-5p	Undetermined	Undetermined
hsa-miR-375	Undetermined	Undetermined
hsa-miR-376c	Undetermined	Undetermined
hsa-miR-503	Undetermined	Undetermined
hsa-miR-193b	Undetermined	Undetermined
hsa-miR-195*	Undetermined	Undetermined
hsa-miR-200b	Undetermined	Undetermined

hsa-miR-214	Undetermined	Undetermined
hsa-miR-340	Undetermined	Undetermined
hsa-miR-381	Undetermined	Undetermined
hsa-miR-518b	Undetermined	Undetermined
hsa-miR-518f	Undetermined	Undetermined
hsa-miR-574-3p	Undetermined	Undetermined
hsa-miR-628-5p	Undetermined	Undetermined
hsa-miR-758	Undetermined	Undetermined
hsa-miR-885-5p	Undetermined	Undetermined

Suppl. Table 3. List of 21 miRNAs measured by TaqMan-based qPCR in volunteers treated with ticagrelor (n = 10), clopidogrel (n = 8) or no drug (n = 8) before (0h) and after (6 h, 24 h) endotoxin administration. RQ: relative quantity.

miRNA	Treatment	Time (pre)	Time (post)	Median RQ (pre)	Median RQ (post)	Median Difference	Difference in Medians	Test Statistic	Effect	P-value	FDR-adjusted p-value
hsa-let7g	Clopidogrel	6h	24h	0.25	0.19	-0.02	-0.06	1.48	0.47	0.16	0.46
		0h	6h	0.25	0.25	0.03	0.01	-0.66	-0.21	0.56	0.86
	Ticagrelor	6h	24h	0.28	0.33	-0.01	0.05	0.56	0.18	0.63	0.91
		0h	6h	0.41	0.28	-0.01	-0.14	0.87	0.27	0.43	0.74
	Untreated	6h	24h	0.28	0.25	-0.05	-0.04	0.36	0.11	0.77	0.98
		0h	6h	0.46	0.28	-0.11	-0.18	0.97	0.31	0.38	0.70
hsa-miR-125b	Clopidogrel	6h	24h	0.77	0.45	-0.37	-0.33	2.40	0.76	0.01	0.10
		0h	6h	0.71	0.77	0.03	0.07	0.25	0.08	0.85	0.98
	Ticagrelor	6h	24h	0.46	0.51	0.04	0.05	-0.97	-0.31	0.38	0.70
		0h	6h	0.93	0.46	-0.46	-0.48	2.80	0.89	0.00	0.02
	Untreated	6h	24h	0.75	0.48	0.08	-0.27	0.25	0.08	0.85	0.98
		0h	6h	1.08	0.75	-0.02	-0.33	0.87	0.27	0.43	0.74
hsa-miR-126	Clopidogrel	6h	24h	0.55	0.49	0.02	-0.06	0.25	0.08	0.85	0.98
		0h	6h	0.54	0.55	-0.07	0.01	0.46	0.15	0.70	0.92
	Ticagrelor	6h	24h	0.52	0.55	0.07	0.04	-0.46	-0.15	0.70	0.92
		0h	6h	0.58	0.52	-0.20	-0.07	2.40	0.76	0.01	0.10
	Untreated	6h	24h	0.41	0.52	0.02	0.11	-0.66	-0.21	0.56	0.86

		0h	6h	0.67	0.41	-0.15	-0.26	2.40	0.76	0.01	0.10
hsa-miR-126STAR	Clopidogrel	6h	24h	7.70	7.87	0.50	0.17	-0.36	-0.11	0.77	0.98
		0h	6h	9.10	7.70	-1.59	-1.40	0.87	0.27	0.43	0.74
	Ticagrelor	6h	24h	5.94	7.57	0.22	1.63	-0.56	-0.18	0.63	0.91
		0h	6h	9.61	5.94	-2.14	-3.67	1.48	0.47	0.16	0.46
	Untreated	6h	24h	7.48	8.08	0.62	0.60	-0.25	-0.08	0.85	0.98
		0h	6h	9.74	7.48	-1.02	-2.26	1.48	0.47	0.16	0.46
hsa-miR-127-3p	Clopidogrel	6h	24h	0.29	0.30	-0.03	0.01	-0.36	-0.11	0.77	0.98
		0h	6h	0.35	0.29	-0.16	-0.06	0.87	0.27	0.43	0.74
	Ticagrelor	6h	24h	0.38	0.74	0.23	0.37	-2.29	-0.73	0.02	0.13
		0h	6h	0.57	0.38	-0.23	-0.20	1.17	0.37	0.28	0.61
	Untreated	6h	24h	0.32	0.49	0.29	0.17	-1.78	-0.56	0.08	0.35
		0h	6h	0.56	0.32	-0.19	-0.24	1.24	0.41	0.25	0.61
hsa-miR-143	Clopidogrel	6h	24h	0.98	0.26	-0.70	-0.73	2.80	0.89	0.00	0.02
		0h	6h	0.45	0.98	0.16	0.53	-0.66	-0.21	0.56	0.86
	Ticagrelor	6h	24h	1.09	0.61	-0.60	-0.49	2.80	0.89	0.00	0.02
		0h	6h	0.57	1.09	0.51	0.52	-1.89	-0.60	0.06	0.29
	Untreated	6h	24h	1.07	0.65	-0.46	-0.41	2.50	0.79	0.01	0.08
		0h	6h	0.60	1.07	0.30	0.47	-2.80	-0.89	0.00	0.02

hsa-miR-146a	Clopidogrel	6h	24h	0.35	0.47	0.08	0.11	-1.48	-0.47	0.16	0.46
		0h	6h	0.47	0.35	-0.11	-0.12	1.78	0.56	0.08	0.35
	Ticagrelor	6h	24h	0.35	0.40	0.10	0.05	-2.09	-0.66	0.04	0.20
		0h	6h	0.44	0.35	-0.05	-0.09	1.48	0.47	0.16	0.46
	Untreated	6h	24h	0.28	0.41	0.08	0.13	-2.19	-0.69	0.03	0.16
		0h	6h	0.49	0.28	-0.14	-0.21	2.50	0.79	0.01	0.08
hsa-miR-150	Clopidogrel	6h	24h	0.19	0.34	0.09	0.15	-2.80	-0.89	0.00	0.02
		0h	6h	0.36	0.19	-0.16	-0.16	2.70	0.85	0.00	0.04
	Ticagrelor	6h	24h	0.26	0.40	0.13	0.14	-2.29	-0.73	0.02	0.13
		0h	6h	0.42	0.26	-0.14	-0.16	2.70	0.85	0.00	0.04
	Untreated	6h	24h	0.25	0.42	0.20	0.16	-2.80	-0.89	0.00	0.02
		0h	6h	0.39	0.25	-0.12	-0.14	2.80	0.89	0.00	0.02
hsa-miR-16	Clopidogrel	6h	24h	0.59	0.36	-0.13	-0.23	1.68	0.53	0.11	0.39
		0h	6h	0.46	0.59	-0.02	0.13	-0.05	-0.02	1.00	1.00
	Ticagrelor	6h	24h	0.18	0.23	-0.01	0.05	1.07	0.34	0.32	0.66
		0h	6h	0.27	0.18	-0.01	-0.09	0.56	0.18	0.63	0.91
	Untreated	6h	24h	0.36	0.12	0.00	-0.24	0.25	0.08	0.85	0.98
		0h	6h	0.23	0.36	-0.02	0.13	0.76	0.24	0.49	0.80
hsa-miR-191	Clopidogrel	6h	24h	0.47	0.33	-0.10	-0.14	2.40	0.76	0.01	0.10
		0h	6h	0.37	0.47	0.09	0.10	-2.09	-0.66	0.04	0.20

	Ticagrelor	6h	24h	0.44	0.37	-0.08	-0.07	1.89	0.60	0.06	0.29
		0h	6h	0.38	0.44	0.08	0.06	-1.48	-0.47	0.16	0.46
	Untreated	6h	24h	0.39	0.35	0.02	-0.04	0.25	0.08	0.85	0.98
		0h	6h	0.40	0.39	-0.03	-0.01	0.46	0.15	0.70	0.92
hsa-miR-197	Clopidogrel	6h	24h	0.56	0.29	-0.26	-0.26	2.80	0.89	0.00	0.02
		0h	6h	0.26	0.56	0.31	0.30	-2.80	-0.89	0.00	0.02
	Ticagrelor	6h	24h	0.56	0.37	-0.21	-0.18	2.80	0.89	0.00	0.02
		0h	6h	0.29	0.56	0.26	0.27	-2.80	-0.89	0.00	0.02
	Untreated	6h	24h	0.53	0.43	-0.11	-0.10	2.80	0.89	0.00	0.02
		0h	6h	0.35	0.53	0.19	0.18	-2.80	-0.89	0.00	0.02
hsa-miR-20b	Clopidogrel	6h	24h	0.68	0.50	-0.19	-0.18	1.68	0.53	0.11	0.39
		0h	6h	0.54	0.68	-0.04	0.14	-0.25	-0.08	0.85	0.98
	Ticagrelor	6h	24h	0.28	0.33	-0.02	0.05	0.87	0.27	0.43	0.74
		0h	6h	0.42	0.28	-0.03	-0.14	0.97	0.31	0.38	0.70
	Untreated	6h	24h	0.36	0.28	0.01	-0.09	0.36	0.11	0.77	0.98
		0h	6h	0.39	0.36	-0.04	-0.03	0.87	0.27	0.43	0.74
hsa-miR-21	Clopidogrel	6h	24h	0.94	0.86	-0.05	-0.08	1.27	0.40	0.23	0.58
		0h	6h	0.87	0.94	-0.08	0.07	-0.05	-0.02	1.00	1.00
	Ticagrelor	6h	24h	0.63	0.65	0.00	0.02	-0.05	-0.02	1.00	1.00
		0h	6h	0.79	0.63	-0.09	-0.17	1.48	0.47	0.16	0.46
	Untreated	6h	24h	0.77	0.74	-0.06	-0.03	0.56	0.18	0.63	0.91

		0h	6h	0.67	0.77	-0.02	0.09	0.87	0.27	0.43	0.74
hsa-miR-221	Clopidogrel	6h	24h	0.59	0.67	-0.01	0.07	-0.15	-0.05	0.92	1.00
		0h	6h	0.66	0.59	-0.13	-0.07	1.68	0.53	0.11	0.39
	Ticagrelor	6h	24h	0.59	0.64	0.11	0.05	-1.78	-0.56	0.08	0.35
		0h	6h	0.57	0.59	-0.13	0.03	1.27	0.40	0.23	0.58
	Untreated	6h	24h	0.44	0.61	-0.02	0.17	-0.25	-0.08	0.85	0.98
		0h	6h	0.58	0.44	-0.09	-0.14	1.27	0.40	0.23	0.58
hsa-miR-223	Clopidogrel	6h	24h	0.85	0.39	-0.32	-0.46	2.80	0.89	0.00	0.02
		0h	6h	0.31	0.85	0.43	0.54	-2.80	-0.89	0.00	0.02
	Ticagrelor	6h	24h	0.83	0.48	-0.33	-0.35	2.80	0.89	0.00	0.02
		0h	6h	0.36	0.83	0.50	0.47	-2.80	-0.89	0.00	0.02
	Untreated	6h	24h	0.84	0.53	-0.22	-0.31	2.80	0.89	0.00	0.02
		0h	6h	0.52	0.84	0.24	0.32	-2.80	-0.89	0.00	0.02
hsa-miR-223STAR	Clopidogrel	6h	24h	17.81	4.75	-11.41	-13.06	2.80	0.89	0.00	0.02
		0h	6h	3.68	17.81	13.46	14.13	-2.80	-0.89	0.00	0.02
	Ticagrelor	6h	24h	18.10	6.92	-10.19	-11.17	2.80	0.89	0.00	0.02
		0h	6h	5.23	18.10	14.44	12.86	-2.80	-0.89	0.00	0.02
	Untreated	6h	24h	16.60	6.90	-8.51	-9.71	2.80	0.89	0.00	0.02
		0h	6h	4.63	16.60	11.36	11.97	-2.80	-0.89	0.00	0.02
		6h	24h	0.69	0.54	-0.16	-0.14	2.60	0.82	0.01	0.05

hsa-miR-24	Clopidogrel	0h	6h	0.45	0.69	0.13	0.23	-2.80	-0.89	0.00	0.02
	Ticagrelor	6h	24h	0.70	0.55	-0.11	-0.15	2.50	0.79	0.01	0.08
		0h	6h	0.50	0.70	0.19	0.20	-2.60	-0.82	0.01	0.05
	Untreated	6h	24h	0.59	0.54	-0.05	-0.06	1.07	0.34	0.32	0.66
		0h	6h	0.51	0.59	0.04	0.08	-1.58	-0.50	0.13	0.45
hsa-miR-26b	Clopidogrel	6h	24h	0.78	0.56	-0.19	-0.22	1.78	0.56	0.08	0.35
		0h	6h	0.53	0.78	-0.03	0.24	-0.56	-0.18	0.63	0.91
	Ticagrelor	6h	24h	0.46	0.43	-0.06	-0.04	1.17	0.37	0.28	0.61
		0h	6h	0.48	0.46	-0.03	-0.02	0.05	0.02	1.00	1.00
	Untreated	6h	24h	0.45	0.38	-0.03	-0.07	0.87	0.27	0.43	0.74
		0h	6h	0.57	0.45	-0.02	-0.12	0.76	0.24	0.49	0.80
hsa-miR-28-3p	Clopidogrel	6h	24h	0.42	0.41	-0.03	-0.01	0.87	0.27	0.43	0.74
		0h	6h	0.35	0.42	0.10	0.07	-1.17	-0.37	0.28	0.61
	Ticagrelor	6h	24h	0.42	0.42	-0.06	0.00	1.17	0.37	0.28	0.61
		0h	6h	0.41	0.42	0.07	0.01	-0.97	-0.31	0.38	0.70
	Untreated	6h	24h	0.34	0.36	0.05	0.03	-1.48	-0.47	0.16	0.46
		0h	6h	0.54	0.34	-0.09	-0.20	2.09	0.66	0.04	0.20
hsa-miR-29a	Clopidogrel	6h	24h	1.12	0.52	-0.49	-0.59	2.80	0.89	0.00	0.02
		0h	6h	0.49	1.12	0.49	0.63	-2.80	-0.89	0.00	0.02
	Ticagrelor	6h	24h	1.01	0.61	-0.35	-0.41	2.70	0.85	0.00	0.04

		0h	6h	0.56	1.01	0.52	0.45	-2.70	-0.85	0.00	0.04
	Untreated	6h	24h	0.86	0.70	-0.20	-0.16	2.19	0.69	0.03	0.16
		0h	6h	0.69	0.86	0.16	0.17	-2.29	-0.73	0.02	0.13
hsa-miR-320a	Clopidogrel	6h	24h	0.62	0.52	-0.10	-0.10	1.27	0.40	0.23	0.58
		0h	6h	0.72	0.62	-0.06	-0.09	0.46	0.15	0.70	0.92
	Ticagrelor	6h	24h	0.36	0.43	0.00	0.07	0.25	0.08	0.85	0.98
		0h	6h	0.49	0.36	-0.05	-0.12	1.17	0.37	0.28	0.61
	Untreated	6h	24h	0.39	0.31	0.02	-0.08	-0.05	-0.02	1.00	1.00
		0h	6h	0.40	0.39	-0.06	-0.02	0.87	0.27	0.43	0.74

Suppl. Table 4. Clinical characteristics of the cohort of sepsis patients. Data is presented as mean ± standard deviation. ANOVA was used to calculate p-values. BMI: body mass index, APACHE II: Acute Physiology And Chronic Health Evaluation II, ALT: alanine aminotransferase, CRP: C-reactive protein.

Parameters	Sepsis patients (n=41)	Survivors (n=30)	Non-survivors (n=11)	P-value
Males (n, %)	26, 70.7%	20, 66.7%	6, 54.5%	
Age (years)	63.37 ± 15.74	58.13 ± 13.77	77.64 ± 11.71	<0.001
BMI (Kg/m2)	25.71 ± 5.08	26.48 ± 5.21	23.43 ± 4.11	0.075
APACHE II Score	18.63 ± 5.88	16.77 ± 4.11	23.73 ± 7.10	0.003
Temperature (ºC)	36.58 ± 1.54	36.75 ± 1.60	36.11 ± 1.32	0.237
Pulse	116.95 ± 22.26	118.13 ± 21.70	113.73 ± 24.50	0.919
Days in hospital	21.93 ± 17.80	21.40 ± 16.92	23.36 ± 20.86	0.965
**Blood tests at enrolment**				
Albumin	25.41 ±6.57	26.70 ± 6.90	21.91 ± 4.01	0.022
Bilirubin	10.24 ±8.50	8.08 ±5.36	17.25 ±13.57	0.078
ALT	63.00± 127.38	75.34 ± 148.10	30.45 ±15.85	0.976
Creatinine	132.29 ±78.09	130.30 ±75.68	137.73 ±87.97	0.977
CRP	212.37 ± 111.62	214.27 ±109.41	207.18 ±122.81	0.691
White blood cells	15.91± 8.05	15.44 ±7.21	17.18 ± 10.27	0.883
Neutrophils	14.30 ±7.70	13.67 ±6.74	16.02 ± 10.05	0.638
Lymphocytes	0.95 ±0.62	1.08 ± 0.66	0.61 ± 0.35	0.015
Monocytes	0.67 ± 0.33	0.74 ± 0.34	0.43± 0.17	0.062

Suppl. Table 5. List of 18 miRNAs measured by TaqMan-based qPCR in sepsis patients at day 1 (d1), day 3 (d3) and day 7 (d7) after admission to the intensive care unit. RQ: relative quantity.

miRNA and day after admission	Survivors (Mean RQ)	Non-Survivors (Mean RQ)	Fold Change	P-value
miR150_d1	4.09	1.34	3.06	0.09
**miR150_d3**	**4.22**	**2.11**	**2.00**	**0.03**
**miR150_d7**	**4.47**	**1.85**	**2.42**	**0.05**
miR320a_d1	1.66	1.57	1.06	0.87
miR320a_d3	1.66	0.67	2.50	0.24
miR320a_d7	1.44	0.88	1.64	0.30
miR20a_d1	3.17	2.29	1.39	0.34
miR20a_d3	3.16	1.58	2.00	0.09
miR20a_d7	3.57	1.87	1.91	0.41
miR21_d1	2.30	4.05	0.57	0.31
miR21_d3	2.12	3.70	0.57	0.39
miR21_d7	3.27	2.01	1.62	0.28
miR24_d1	2.53	1.77	1.43	0.21
miR24_d3	2.37	1.55	1.53	0.22
miR24_d7	2.43	1.33	1.83	0.24
miR146_d1	6.41	3.78	1.70	0.36
miR146_d3	4.70	1.89	2.48	0.10
miR146_d7	3.98	2.24	1.78	0.33
miR191_d1	5.63	3.39	1.66	0.25
miR191_d3	4.69	2.98	1.58	0.25
miR191_d7	6.58	2.68	2.45	0.55
miR197_d1	2.00	0.72	2.77	0.43
miR197_d3	0.93	0.64	1.46	0.27
miR197_d7	1.26	0.58	2.16	0.36
miR16_d1	3.13	2.19	1.43	0.30
miR16_d3	2.72	1.89	1.44	0.335
miR16_d7	2.42	2.48	0.97	0.96
miR126_d1	2.73	1.96	1.39	0.43
miR126_d3	2.44	1.89	1.29	0.34

miR126_d7	2.36	1.40	1.69	0.23
miR223_d1	3.01	1.83	1.65	0.51
miR223_d3	3.07	1.20	2.55	0.36
miR223_d7	1.35	0.92	1.46	0.65
miR155_d1	0.72	0.77	0.94	0.56
miR155_d3	0.62	0.72	0.86	0.61
miR155_d7	0.64	0.65	0.98	0.68
miR26b_d1	9.00	3.02	2.98	0.362
miR26b_d3	4.99	9.62	0.52	0.20
miR26b_d7	4.51	4.37	1.03	0.97
miR125b_d1	5.67	16.48	0.34	0.14
miR125b_d3	3.15	60.11	0.05	0.10
miR125b_d7	13.77	16.38	0.84	0.85
miR-143_d1	12.81	33.55	0.38	0.20
miR-143_d3	18.73	5.75	3.26	0.39
miR-143_d7	18.46	4.63	3.98	0.16
miR-133_d1	2.28	8.24	0.28	0.08
miR-133_d3	6.53	2.90	2.25	0.58
miR-133_d7	3.49	0.85	4.11	0.17
miR122_d1	25.53	13.01	1.96	0.41
miR122_d3	17.85	7.02	2.54	0.10
miR122_d7	37.36	77.06	0.48	0.16
miR185_d1	49.14	18.45	2.66	0.52
miR185_d3	9.58	4.12	2.32	0.43
miR185_d7	8.94	11.99	0.75	0.65
